# FOXO3a Protects against Kidney Injury in Type II Diabetic Nephropathy by Promoting Sirt6 Expression and Inhibiting Smad3 Acetylation

**DOI:** 10.1155/2021/5565761

**Published:** 2021-05-26

**Authors:** Xiaowei Wang, Tingting Ji, Xiaoying Li, Xiaolei Qu, Shoujun Bai

**Affiliations:** ^1^Department of Endocrinology, Qingpu Branch of Zhongshan Hospital Affiliated to Fudan University, 1158 Gongyuan East Road, Qingpu District, Shanghai 201700, China; ^2^Department of Nephrology, Qingpu Branch of Zhongshan Hospital Affiliated to Fudan University, 1158 Gongyuan East Road, Qingpu District, Shanghai 201700, China

## Abstract

Diabetic nephropathy (DN) is the most common cause of end-stage renal disease. Although numerous reports have demonstrated a correlation between epithelial-mesenchymal transition (EMT) and renal fibrosis, how these processes lead to tubular dysfunction remains unclear. Here, we show that FOXO3a protects kidneys from injury in type II DN by increasing Sirt6 expression, which deacetylates Smad3 and inhibits its transcriptional activity. The results showed that progressive EMT in the kidneys from db/db mice is associated with Sirt6 downregulation and involved in tubular injury and dysfunction. The reduction of Sirt6 levels in db/db mice resulted in progressive kidney injury, indicating the protective role of Sirt6. Furthermore, Sirt6 was shown to directly bind to Smad3, a key downstream mediator of TGF-*β*, and could deacetylate it to inhibit its nuclear accumulation and transcriptional activity in HK2 cells. Besides, we demonstrate that FOXO3a activates Sirt6 expression by binding to its promoter. shRNA-induced FOXO3a knockdown in the kidneys of db/db mice exacerbated tubular injury and renal function loss. Mechanistically, FOXO3a protects against kidney injury in type II DN through the Sirt6/Smad3 axis. Thus, the pharmacological targeting of FOXO3a-mediated Sirt6/Smad3 signaling pathways may provide a novel strategy for treating type II DN.

## 1. Introduction

Diabetic nephropathy (DN) is a type of end-stage renal disease that accounts for approximately 16.4% of all diabetes cases in China [1] and afflicts more than 20-40% of patients with diabetes globally [2]. Although glomerular lesions are the major cause of DN, many studies have demonstrated that tubular injury, which is attributable to epithelial-mesenchymal transition (EMT) [3, 4], has a significant role in DN [5–11]. However, the understanding of the underlying pathogenesis of tubular injury in DN remains incomplete.

Several reports have shown that transforming growth factor-*β* (TGF-*β*)/Smad3 signaling pathways contribute to EMT and renal fibrosis [12–15], with most having focused on the role of Smad3 phosphorylation in the EMT process in DN. However, little research has been performed concerning the role of Smad3 acetylation in the EMT of DN, which also plays a significant role in the EMT in cancers, such as lung cancer [16], nasopharyngeal carcinoma [17], breast cancer [18], and other cancer cells [19].

Sirt6 (gene ID: 51548), also known as sirtuin 6 or SIR2L6, is a member of the sirtuin family of class III NAD+-dependent deacetylases, which affect the physiology of multiple cell types, tissues, and phenotypes associated with aging, cancer, metabolic disorders, and liver fibrosis [20–23]. Recently, Liu et al. showed that Sirt6 plays a protective role in podocyte injury and kidney proteinuria, indicating the importance of Sirt6 in kidney disease [24]. However, little is known regarding the role of Sirt6 in DN.

Therefore, the goal of the present study was to elucidate the role of Sirt6 in EMT and tubular injury caused by DN. We observed lower Sirt6 expression in high glucose- (HG-) induced HK2 cells and in the kidneys of db/db mice (type II diabetes model). Sirt6 knockdown aggravated kidney injury associated with DN both in vitro and in vivo. We observed that FOXO3a exhibited the same function in EMT and tubular injury in DN. Mechanistically, FOXO3a is responsible for promoting Sirt6 expression, which deacetylates Smad3, resulting in the reduction of Smad3 in the nucleus, suppression of EMT, and exacerbation of tubular injury. Our findings indicate that Sirt6 might be a potential therapeutic target in kidney diseases such as DN.

## 2. Materials and Methods

### 2.1. Cell Culture

The HK2 cell line was obtained from the China Center for Type Culture Collection (CCTCC). HK2 cells were cultured in MEM (containing 5 mM D-glucose) (Gibco) supplemented with 10% FBS (Gibco), 100 U/ml penicillin, and 100 *μ*g/ml streptomycin (Gibco) at 37°C under an atmosphere with 5% CO_2_. HK2 cells were cultured in the HG medium (containing 30 mM D-glucose) for 72 h to induce EMT. In comparison, control cells were cultured in a medium with a high concentration of mannitol (Mann) (5 mM D-glucose and 25 mM mannitol). The cells used in the experiments were all from the first 10 generations.

### 2.2. CRISPR/Cas9-Induced Smad3 Knockout

Cas9-GFP protein and sgRNA were obtained from GenScript, where the sgRNA sequence (5′-GCGGCTCTACTACATCGGAG-3′) targeted exon 6 of the Smad3 gene. The CRISPR/Cas9 system was used as described in a previous study [25]. Briefly, before transfection, HK2 cells (2.5 × 10^5^ cells per well) were seeded in a 6-well plate. Then, Opti-MEM (Gibco) containing the Lipofectamine Cas9 Plus™ Reagent (Invitrogen) was used to transfect a mixture of 50 pmol Cas9-GFP protein and 50 pmol sgRNA into HK2 cells, which were cultured for 2 days at 37°C. Then, GFP-positive cells were sorted using a FACSAria instrument (BD Biosciences) and seeded into a 96-well plate using the limiting dilution method. Single clones were obtained after 3 weeks and identified by sequencing and western blot analyses. Cells with successful Smad3 knockout (HK2-Smad3^−/−^) were used for subsequent experiments.

### 2.3. Transfection and Transduction

Small interfering RNAs (siRNAs) targeting Sirt6 (si-Sirt6) or FOXO3a (si-FOXO3a) or used for the negative control (si-NC) were purchased from Sangon Biotech. The Lipofectamine 2000 reagent was used to perform transfection experiments according to the manufacturer's instructions (Thermo Fisher Scientific).

For generating stable overexpression cell lines, lentiviruses encoding wild-type Smad3 (WT-Smad3) or K333A and K378A mutant Smad3 (Mut-Smad3) were transduced into HK2-Smad3^−/−^ cells and screened with 2 *μ*g/ml of puromycin (Sangon Biotech) for 2 weeks. Lentiviruses were packaged in HEK293FT cells cotransfected with pCDH-CMV-Mcs-Ef1-Puro, psPAX2, and pMD2G (Addgene) for 72 h. The sequences of siRNAs and primers (Sangon Biotech) are shown in Table S1.

### 2.4. Animal Experiments

7-8-week-old male type II diabetic db/db mice and genetic control db/+ mice were obtained from the Jackson Laboratory. Lentivirus was injected when the mice were eighteen weeks old. For each mouse, 50 *μ*l of ultracentrifuge-purified lentivirus contains small hairpin RNA (shRNA) targeting Sirt6 (sh-Sirt6), FOXO3a (sh-FOXO3a), or none (si-NC). Lentivirus (10 *μ*g/ml P24) was directly microinjected into the kidney. Briefly, mice were anesthetized with isoflurane and incisions less than 1 cm were cut from both sides of the back of the mice to expose kidneys, and 4 injection sites were selected in the cortex of each kidney. Enrofloxacin (2.5 mg/kg) and buprenorphine (0.05 mg/kg) were injected subcutaneously before the surgery and every 8-12 h for 48 h after surgery. The body temperature of the mice was controlled at 37 ± 0.5°C throughout the surgery using a heating lamp and a self-monitored heating pad. Mice were housed under controlled temperature conditions (20°C) and humidity (60%) with a 12-hour light/dark cycle and had free access to food and water.

Fasting blood glucose levels from the tail vein were determined weekly by the Glucose Assay Kit (Abcam) to show that the groups of mice were matched for the severity of diabetes at the start of the experiment, and lentivirus injection did not affect blood glucose levels (Fig. S1a-c). The 6 h fast lasted from 7 a.m. to 1 p.m. with blood drawn from the tail vein at 1 p.m., only provided access to water. In our study, a fasting blood glucose level of >20 mmol/l was regarded as diabetic.

Kidneys from mice were obtained two weeks after lentivirus injection. The kidney samples from mice with successful target gene knockdown induced by sh-Sirt6 or sh-FOXO3a (analyzed by western blot) and without obvious toxicity were used in subsequent experiments.

All methods were performed following the guidelines and regulations of the Zhongshan Hospital Affiliated to Fudan University Animal Care and Use Committee and the National Institutes of Health.

### 2.5. Luciferase Assays

For Smad-binding element- (SBE-) Luc reporter assays, HK2 or HK2-Smad3^−/−^ cells were seeded into 48-well plates and transiently cotransfected with an SBE luciferase reporter (Genomeditech, China) and Sirt6 or si-Sirt6 as previously described [26]. In some experiments, OSS_128167 (Sigma) was added after 24 h of transfection at different concentrations. After 48 h of transfection, luciferase activity was detected.

To analyze the binding ability of FOXO3a to the Sirt6 promoter, the -2083 to -1859 region of the Sirt6 promoter was PCR-amplified using DNA from HK2 cells as a template [27]. The mutant Sirt6 promoter was obtained from Sangon Biotech, and both the wild-type and mutant Sirt6 promoter sequences were separately inserted into the pGL3-basic luciferase reporter vector (Promega Corporation). Then, the pGL3 vector, pRL-TK, and si-FOXO3a or FOXO3a were transiently cotransfected into HK2 cells. After 48 h of transfection, luciferase activity was detected using a Dual-Lumi™ Luciferase Reporter Gene Assay Kit (Beyotime Institute of Biotechnology) [27] and was normalized to *Renilla* luciferase activity. All primers are shown in Table S1.

### 2.6. Coimmunoprecipitation (co-IP)

HK2 cells were lysed with the NP-40 lysis buffer (50 mM Tris (pH 7.5), 150 mM NaCl, 1 mM EDTA, and 0.25% sodium deoxycholate) containing 1 mM phenylmethylsulfonyl fluoride (PMSF) (Sigma) and protease inhibitor cocktail (Sangon Biotech). Proteins were incubated with anti-Flag (Abcam) and Protein A/G PLUS-Agarose (Santa Cruz Biotechnology) at 4°C overnight. Then, the protein-bound beads were washed with the lysis buffer and then denatured in the SDS buffer (0.1 M Tris-HCl (pH 6.8), 4% SDS, 20% glycerol, sodium pyrophosphate, 1 mM Na_3_VO_4_, and NaF) at 100°C for 10 min. SDS-PAGE analyzed cell extracts and immunoprecipitated proteins. The endogenous co-IP was performed similarly with the above excepting the following points: (1) proteins from the kidney were extracted by using the T-PER™ Tissue Protein Extraction Reagent (Thermo Fisher Scientific) containing 1 mM PMSF; and (2) proteins were incubated with anti-Smad3 antibodies (Abcam).

### 2.7. Western Blot Analysis

Proteins from cells were extracted with the RIPA buffer (Beyotime Institute of Biotechnology), while those from mouse kidneys were extracted using a One Step Animal Tissue Active Protein Extraction Kit (Sangon Biotech). Subsequently, the proteins were denatured at 100°C for 10 min and then separated by 4-20% SDS-PAGE (Bio-Rad) at 120 V for 90 min before being transferred to nitrocellulose membranes (Boster) at 300 A for 1.5 h. The membranes were incubated with primary antibodies overnight at 4°C after being blocked with 5% fat-free milk for 1 h. After washing, the membranes were incubated with secondary antibodies for 2 h at room temperature and treated with a high-signal electrochemiluminescence kit (Fdbio Science) and then detected using an image analysis system (Image-Pro Plus 6.0, Media Cybernetics). Anti-*α*-SMA (1 : 1000), anti-Smad3 (1 : 1000), anti-E-cadherin (1 : 500), anti-Sirt6 (1 : 1000), anti-KIM-1 (1 : 1000), anti-NGAL (1 : 1000), and anti-Collagen I (1 : 1000) were obtained from Abcam. Anti-Acetylated-Lysine Antibody (1 : 1000) was obtained from Cell Signaling Technology. Anti-GAPDH (1 : 5000) and anti-Lamin B (1 : 5000) were obtained from SinoBiolohical. All second HRP antibodies (1 : 5000) were obtained from Sangon Biotech.

### 2.8. Histology and Immunohistochemistry (IHC) Staining

Kidneys from mice two weeks after lentivirus injection were fixed in 10% formalin, paraffin-embedded, sectioned into 4 *μ*m thick sections, and stained with the periodic acid-Schiff (PAS) staining kit (Beyotime) for histological analysis. Briefly, paraffin sections dewaxing to water were incubated with periodic acid staining solution for 10 min. Then, the sections were incubated with Hematoxylin Staining Solution for 5 min and 1% hydrochloric acid alcohol differentiation after staining with the Schiff Reagent for 30 min at 37°C in the dark. Next, the sections were dehydrated, vitrified, and sealed after washing by running water.

The statistical analysis of tubular injury was similar to the method performed by Takaori et al. [28]. The pathological characteristics in proximal tubules were the (i) disorder proximal tubular structure, (ii) cellular debris in the lumen of proximal tubules, and (iii) loss of the brush border. The tubular injury score was evaluated according to the following scoring system: 0 = no tubular injury; 1 = 0~10% tubules injured; 2 = 11%–25% tubules injured; 3 = 26%–50% tubules injured; 4 = 51%–74% tubules injured; and 5 = 75~100% tubules injured.

For histochemical staining, paraffin-embedded sections were stained with anti-Sirt6, anti-E-cadherin, anti-*α*-SMA, and anti-FOXO3a antibodies.

### 2.9. Chromatin Immunoprecipitation (ChIP)

ChIP assays were performed using HK2 cells with an EZ-ChIP™ kit (Merck KGaA). Purified DNA was used for RT-PCR. All primers used for ChIP analyses are shown in Table S1.

### 2.10. Quantitative Reverse Transcription PCR (qPCR)

Total RNA was extracted from cells and kidney tissues using an RNeasy Mini Kit (Qiagen) and reverse-transcribed into complementary DNA (Takara). Quantitative PCR was performed using the SYBR green-based assay by the TB Green® Fast qPCR Mix Kit (Takara) with a CFX96 Real-Time system (Bio-Rad).

### 2.11. Mouse Metabolic Measurements

The albumin and creatinine ratio (ACR), blood urea nitrogen (BUN), and proteinuria levels in mice were measured 2 weeks after infection. A mouse albumin ELISA kit (Bethyl Laboratories) and a creatinine assay kit (BioAssay Systems) were used to determine ACR values. A urea assay kit (BioAssay Systems) was used to measure BUN. A proteinuria ELISA kit (Nanjing Anyan Biological Technology) was used to measure proteinuria.

### 2.12. Wound Healing Assay

HK2 cells were seeded in 6-well plates (5 × 10^5^ cells/well). A “wound” was then generated in each well using a 200 *μ*l pipette tip, and the cells were imaged at 0 and 48 h. The wound areas were quantified by ImageJ with the normalized “relative wound area” (wound areas (48 h)/wound areas (0 h)) according to others' work [29, 30].

### 2.13. Transwell Assay

For the Transwell assay, a 24-well insert with an 8 *μ*m pore size and precoated with Matrigel (Corning, 354277) was used according to the manufacturer's directions. Then, a 500 *μ*l medium with 20% FBS was added to the lower side of the Transwell chamber, and 5 × 10^4^ cells in a 250 *μ*l FBS-free medium were loaded into the upper side. After 24 h, cells on the underside of the membrane were fixed with 4% paraformaldehyde (Sangon Biotech, E672002) for 30 min and stained with 0.1% crystal violet solution (Sangon Biotech, E607309) for 30 min and further counted in 5 random fields under a microscope.

### 2.14. Immunofluorescence (IF)

2 × 10^4^ cells/well were seeded on glass slides coated with 0.1% Gelatin (Gibco) in a 24-well plate (5 × 10^4^ cells/well) and cultured in a different medium for 48 h before staining. Then, cells were fixed with 4% paraformaldehyde, permeabilized with 0.1% Triton X-100, blocked with the blocking buffer (1% bovine serum albumin (Sangon Biotech) and 0.02% Triton X-100 (Sigma) in phosphate-buffered saline), and stained with antibodies according to the manufacturer's instructions. The anti-Smad3 antibody was obtained from Abcam. DAPI was obtained from Sangon Biotech. Goat anti-rabbit Alexa Fluor 488 was from Invitrogen.

### 2.15. Statistics

All quantitative data were evaluated for normality of distribution using the Shapiro-Wilk test. Student's *t*-test was performed using Prism (version 5; GraphPad Software). For comparing multiple groups, one-way ANOVA followed by Tukey's multiple comparison test was performed using Prism. The data are presented as the means ± SD. *p* < 0.05 was considered to indicate a statistically significant difference.

## 3. Results

### 3.1. Sirt6 Is Associated with EMT in HG-Induced HK2 Cells

**Figure 1:**
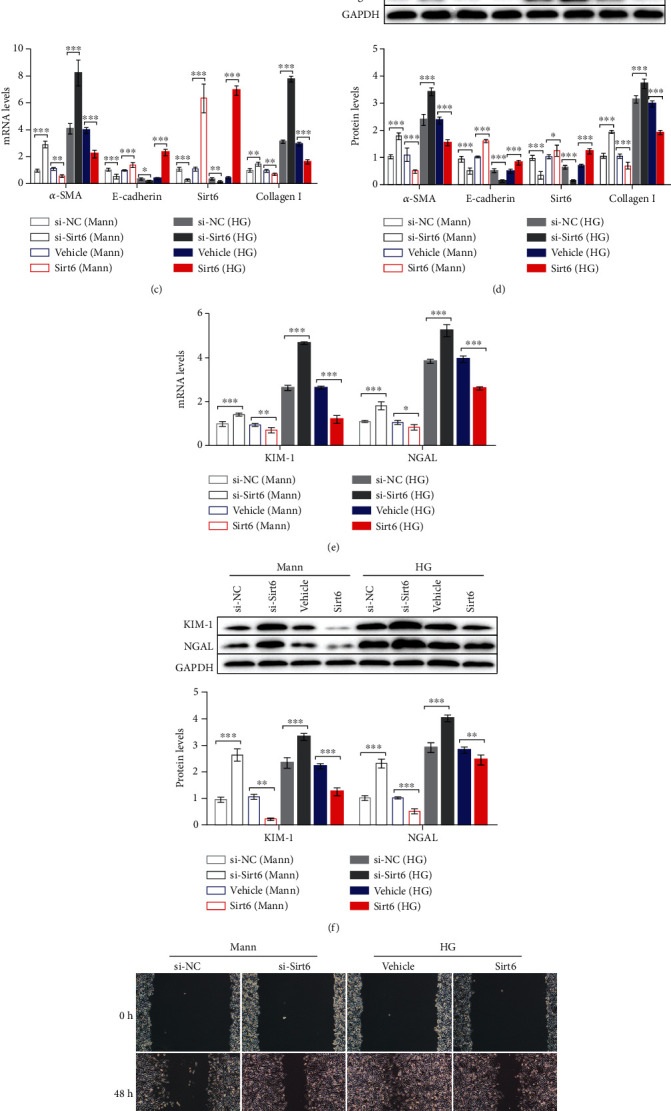
The progression of EMT HG-induced HK2 cells is associated with reduced Sirt6 levels. (a, b) HK2 cells were cultured in the HG medium for 72 h. (a) mRNA expression of fibrotic genes and Sirt6 analyzed by qPCR. (b) Protein expression of fibrotic genes and Sirt6 assessed by western blot analysis. (c–f) HK2 cells were transfected with si-NC or si-Sirt6 to knock down Sirt6 or transfected with the vehicle or the Sirt6 overexpression vector to increase Sirt6 expression and cultured in the Mann medium or HG medium for 72 h. (c) mRNA expression of fibrotic genes and Sirt6 analyzed by qPCR. (d) Protein expression of fibrotic genes and Sirt6 assessed by western blot analysis. (e) mRNA expression of tubular damage genes analyzed by qPCR. (f) Protein expression of tubular damage genes assessed by western blot analysis. (g, h) Representative images of cell migration and graphs showing the wound area quantification in HK2 cells. After 48 h of transfection, wounds were generated using a 200 *μ*l pipette tip and cultured for another 48 h. HK2 cells transfected with si-NC or si-Sirt6 were cultured in the Mann medium, and cells transfected with the vehicle or the Sirt6 overexpression vector were cultured in the HG medium. (i) Representative images of Transwell assay and graphs showing migrated cells. After 24 h of seeding, HK2 cells were transfected with si-NC or si-Sirt6 cultured in the Mann medium and transfected with the vehicle or the Sirt6 overexpression vector cultured in the HG medium. The data are presented as the means ± SD. *n* = 3 experiments in (a–i). ^∗^*p* < 0.05, ^∗∗^*p* < 0.01, and ^∗∗∗^*p* < 0.01.

We first assessed the expression patterns of the EMT markers TGF-*β*, *α*-SMA, Collagen I, and E-cadherin in HK2 cells cultured in the HG and Mann medium. The qPCR and western blot results showed that TGF-*β*, *α*-SMA, and Collagen I levels were increased while E-cadherin expression was reduced (Figures 1(a) and 1(b)), suggesting that HG-induced HK2 cells displayed EMT.

Interestingly, we observed a notable reduction in Sirt6 expression in HG-induced HK2 cells at both the mRNA and protein levels (Figures 1(a) and 1(b)). To determine whether Sirt6 is involved in EMT in HK2 cells, we analyzed the changes in *α*-SMA, Collagen I, and E-cadherin expression in response to changes in Sirt6 expression. Cells were transfected with si-Sirt6 to knock down its expression or with si-NC. The qPCR results showed that *α*-SMA and Collagen I mRNA expression was enhanced, whereas that of E-cadherin was decreased due to an approximately 70% downregulation of Sirt6 mRNA induced by si-Sirt6 compared to that observed in the si-NC group in both the Mann- and HG-induced HK2 cells (Figure 1(c)). The same trend was observed in protein levels observed by western blot analysis (Figure 1(d)). In addition, we cultured HK2 cells overexpressing Sirt6 to investigate the influence of Sirt6 in EMT further. We observed that Sirt6-overexpressing cells showed lower *α*-SMA and Collagen I expression with higher E-cadherin expression than cells transfected with the empty vector control at both the mRNA and protein levels (Figures 1(c) and 1(d)). These results indicated that *α*-SMA and E-cadherin expression alterations were associated with Sirt6 levels in HK2 cells.

Moreover, we analyzed the level of tubular damage-related protein, kidney injury molecule-1 (KIM-1), and neutrophil gelatinase-associated lipocalin (NGAL) [31]. The levels of KIM-1 and NGAL were increased in sh-Sirt6 treatment groups and decreased in Sirt6 overexpression groups, indicating that downregulation of Sirt6 aggravated tubular damage in HK2 cells (Figures 1(e) and 1(f)).

Subsequently, we assessed the influence of Sirt6 on HK2 cell migration induced by the HG or Mann medium, as this phenotype is closely associated with EMT [32]. Mann-induced HK2 cells treated with si-Sirt6 showed a lower relative wound area than those treated with si-NC, indicating that si-Sirt6 promoted the migration of HK2 cells in the Mann medium (Figures 1(g) and 1(h)). In contrast, HG-induced HK2 cells with Sirt6 overexpression exhibited attenuated migration compared to the vehicle groups (Figures 1(g) and 1(h)). Similar results were also observed in the Transwell assay (Figure 1(i)). These findings indicated that Sirt6 could protect HK2 cells against the EMT process.

### 3.2. Downregulation of Sirt6 Aggravates Kidney Injury in db/db Mice

**Figure 2:**
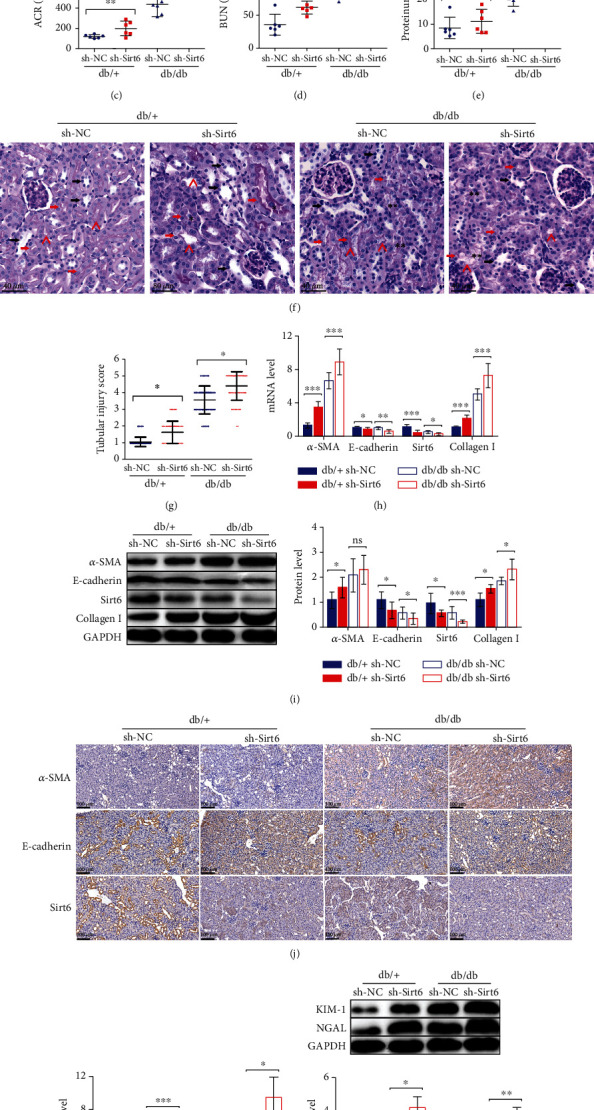
Reduced Sirt6 levels contribute to the exacerbation of kidney injury in db/db mice. (a, b) Protein and mRNA were extracted from the kidneys of 20-week-old db/db and db/+ mice. (a) Sirt6 mRNA expression analyzed by qPCR. (b) Sirt6 protein expression assessed by western blot analysis. (c–j) The db/db or db/+ mice used in all experiments were 20 weeks old and were infected with sh-NC or sh-Sirt6 lentiviruses in the kidneys at 18 weeks old to knock down Sirt6 expression in vivo. (c–e) Graphs showing ACR, BUN, and proteinuria as analyzed by the ELISA kit. (f) Representative images of PAS staining of kidneys from mice. Scale bar, 40 *μ*m. (g) Scoring of tubular injury. Proximal and distal tubules are marked with red and black arrows, respectively. “^” represents the brush border of proximal tubules. “^∗^” represents the cellular debris in the lumen of proximal tubules. “^∗∗^” represents the disorder proximal tubular structure, respectively. (h) *α*-SMA, E-cadherin, Sirt6, and Collagen I mRNA expression analyzed by qPCR. (i) Protein expression of *α*-SMA, E-cadherin, Sirt6, and Collagen I assessed by western blot analysis. (j) IHC analysis of *α*-SMA, E-cadherin, and Sirt6 expression in kidney tissue. (k) KIM-1 and NGAL mRNA expression analyzed by qPCR. (l) Protein expression of KIM-1 and NGAL assessed by western blot analysis. The data are presented as the means ± SD. *n* = 6 experiments in (a–e) and (h–l). *n* = 60 experiments in (g). ^∗^*p* < 0.05, ^∗∗^*p* < 0.01, and ^∗∗∗^*p* < 0.01.

To investigate the role of Sirt6 in EMT and kidney injury in vivo, we used type II diabetic db/db mice and genetic control db/+ mice as our animal models. Decreased Sirt6 levels were observed in the kidneys of db/db mice as assessed by qPCR and western blot analyses (Figures 2(a) and 2(b)) (*p* = 0.0249 in Figure 2(a)).

We also knocked down Sirt6 in mice by injecting lentivirus containing sh-Sirt6 into both kidneys of db/db or db/+ mice. Subsequently, the mice were evaluated for several DN pathological markers, such as the ACR, BUN, and proteinuria. Notably, we observed a greater exacerbation in those pathological characteristics in the kidneys of mice injected with sh-Sirt6 injection in both the db/+ and db/db mice (Figures 2(c)–2(e)), indicating that Sirt6 knockdown in mouse kidneys increased the severity of DN compared to the sh-NC controls.

Periodic acid-Schiff (PAS) staining results revealed tubular injury characteristics with the disorder proximal tubular structure, cellular debris in the lumen of proximal tubules, and loss of the brush border in the kidneys of db/db mice treated with sh-Sirt6 relative to the sh-NC groups (Figure 2(f)) [28, 33]. The tubular injury scoring showed that Sirt6 knockdown induced by sh-Sirt6 promoted the tubular damage (Figure 2(g)). Similar results were observed in db/+ mice treated with sh-Sirt6, although the effect was less pronounced than that detected in db/db mice (Figures 2(f) and 2(g)). Then, we analyzed the expression of EMT markers in the kidneys of mice from the four groups through qPCR and immunoblotting analyses. Sirt6 expression was notably reduced in db/db mice after sh-Sirt6 injection (Figures 2(h) and 2(i)). We observed increased *α*-SMA levels and inhibited E-cadherin expression when Sirt6 was knocked down by sh-Sirt6 in both the db/db and db/+ mice (Figures 2(h) and 2(i)). Regarding the differences in EMT marker expression between the db/db and db/+ mice, much higher *α*-SMA and Collagen I expression and much lower E-cadherin expression were observed in the kidneys of db/db mice, even in db/+ mice treated with sh-Sirt6 (Figures 2(h) and 2(i)). Furthermore, the IHC results showed similar findings (Figure 2(j)). We also observed that the levels of KIM-1 and NGAL in groups treated with sh-Sirt6 were much higher than that in controls (Figures 2(k) and 2(l)).

Together, our results showed that inhibition of Sirt6 expression induced by sh-Sirt6 in the kidneys of db/db and db/+ mice could promote the EMT, aggravate the pathological characteristics of DN, and exacerbate the tubular injury, suggesting that Sirt6 has a protective role in the kidney injury induced by DN.

### 3.3. Sirt6 Protects against the Progression of Kidney Injury by Deacetylating Smad3 in TGF-*β* Signaling

Among the multiple signaling pathways associated with renal tubular EMT and injury, the TGF-*β*/Smad3 pathway is key to triggering the activation of multiple downstream regulators in DN and depends on Smad3 phosphorylation [12–15]. However, some reports have indicated that Smad3 acetylation also activates EMT in several cancers [16–18]. Zhong and colleagues' results indicated that Sirt6 alleviates EMT and liver fibrosis in hepatic stellate cells by deacetylating conserved lysine residues (K333 and K378) on Smad3 [23]. Therefore, we hypothesized that a similar mechanism might be involved in DN-induced kidney injury.

**Figure 3:**
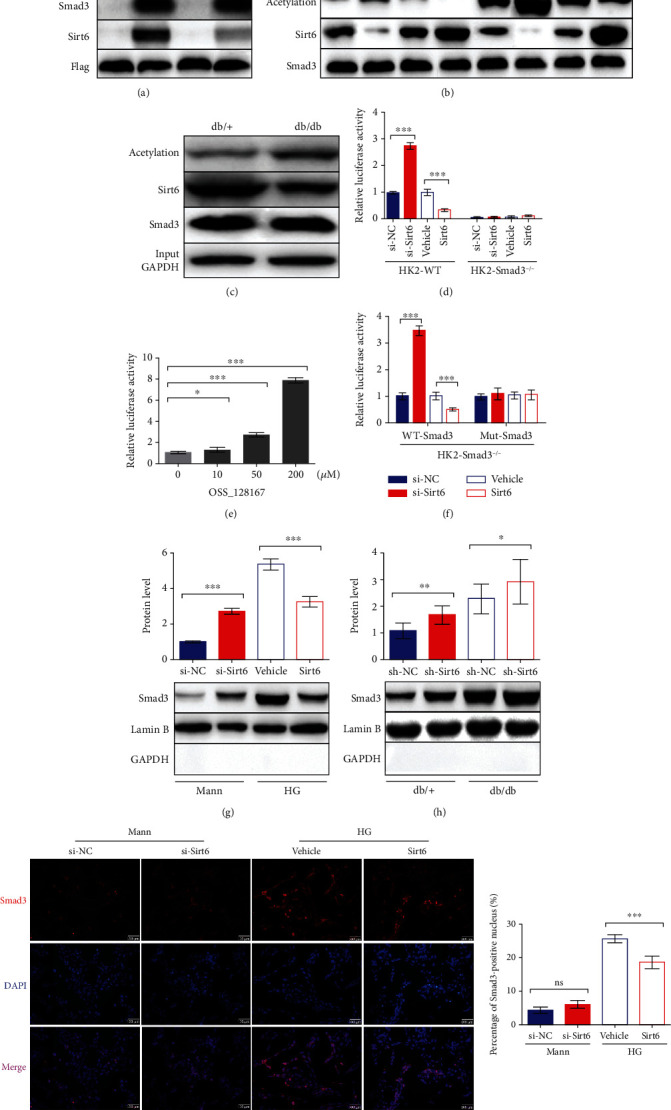
Sirt6 inhibits Smad3 transcriptional activity by deacetylating it and suppressing its nuclear localization. (a, b) Exogenous and endogenous co-IP assays performed to assess the binding and between Sirt6 and Smad3 and Smad3 acetylation in HK2 cells. (a) HK2 cells cultured in the Mann or HG medium were transfected with Flag-Smad3 or Flag-IgG. Proteins immunoprecipitated with an anti-Flag antibody were analyzed using anti-acetylation, anti-Smad3, anti-Sirt6, and anti-Flag antibodies. (b) HK2 cells were cultured in the Mann or HG medium for 72 h. Proteins immunoprecipitated with an anti-Smad3 antibody were analyzed using anti-acetylation, anti-Sirt6, and anti-Smad3 antibodies. (c) Endogenous co-IP analysis to assess the binding and between Sirt6 and Smad3 and Smad3 acetylation in the kidneys of db/+ or db/db mice. Proteins immunoprecipitated with an anti-Smad3 antibody were analyzed using anti-acetylation, anti-Sirt6, and anti-Smad3 antibodies. Proteins from whole-cell lysates analyzed using anti-GAPDH antibodies. (d–f) The luciferase activity for the SBE-Luc reporter assay. (d) Wild-type HK2 cells and HK2-Smad3^−/−^ cells cultured in the Mann medium were cotransfected with SBE-Luc plasmids with the si-NC, si-Sirt6, vehicle, and Sirt6 overexpression vector for 72 h, respectively. (e) Wild-type HK2 cells were transfected with SBE-Luc plasmids for 72 h. Different concentrations of OSS_128167 were added into the Mann medium to inhibit Sirt6 deacetylation at 24 h after transfection. (f) HK2-Smad3^−/−^ cells stably overexpressing wild-type or mutant Smad3 with K333A and K378A mutations were medium-cotransfected with SBE-Luc plasmids with the si-NC, si-Sirt6, vehicle, and Sirt6 overexpression vector, respectively, and cultured in the Mann medium for 72 h. (g, h) Nuclear proteins were extracted from HK2 cells or the kidneys of db/db and db/+ mice. (g) Smad3 protein expression in HK2 cells transfected with the si-NC, si-Sirt6, vehicle, and Sirt6 overexpression plasmid, as assessed by western blot analysis. (h) Smad3 protein expression in the kidneys of db/db or db/+ mice injected with sh-NC or sh-Sirt6 lentivirus, as assessed by western blot analysis. (i) Representative IF images of HK2 cells staining with Smad3 and DAPI in different conditions. The data are presented as the means ± SD. *n* = 3 experiments in (a–g) and (i). *n* = 6 experiments in (h). ^∗^*p* < 0.05, ^∗∗^*p* < 0.01, and ^∗∗∗^*p* < 0.01.

We first assessed the deacetylation activity of Sirt6 against Smad3 through exogenous IP assays. We transfected Flag-tagged Smad3 (Flag-Smad3) into HK2 cells cultured in the Mann or HG medium. After being pulled down with an anti-Flag antibody, we analyzed the endogenous Sirt6 and acetylated Smad3 levels. The results showed higher acetylated Smad3 levels in HG-induced HK2 cells and lower Sirt6 levels following the overexpression of exogenous Smad3 (Figure 3(a)). The reduced Sirt6 levels indicated that Sirt6 directly binds to Smad3 and that Sirt6 levels are decreased in HG-induced HK2 cells, consistent with previous results. To further confirm these findings, we performed endogenous IP assays to investigate the changes in acetylated Smad3 levels resulting from Sirt6 silencing with si-Sirt6 or Sirt6 overexpression. We treated cells with si-NC or si-Sirt6 and assessed the Smad3 acetylation using an anti-acetylation antibody after Smad3 pulldown. The results indicated that Sirt6 downregulation increased acetylated Smad3 levels in both the Mann and HG groups (Figure 3(b)). In contrast, IP results showed that the acetylated Smad3 levels dramatically decreased in cells overexpressing Sirt6 (Figure 3(b)). Furthermore, we assessed Smad3 acetylation in the kidneys of db/+ or db/db mice by IP and observed higher levels of acetylated Smad3 in the kidneys of db/db mice than in db/+ mice (Figure 3(c)). These results indicate that Sirt6 can deacetylate Smad3 in vitro and in vivo.

Subsequently, we investigated whether changes in Smad3 acetylation induced by Sirt6 affect its downstream signaling. To this end, we performed SBE-Luc reporter assays to assess the activation of Smad3 downstream signaling [16]. We transiently cotransfected HK2 cells cultured in the Mann medium with SBE-Luc reporter plasmids and si-Sirt6 or the Sirt6 overexpression plasmid. Luciferase activity was increased in the presence of si-Sirt6 and inhibited by Sirt6 overexpression (Figure 3(d)). We also generated HK2-Smad3^−/−^ cells using the CRISPR/Cas9 system to verify the reporter results and observed no luciferase activity, even in cells silenced with si-Sirt6 or those overexpressing Sirt6 (Figure 3(d)). To confirm these results, we assessed the effect of the micromolecule OSS_128167, a Sirt6-specific inhibitor [34], in an SBE reporter assay. As the concentration of OSS_128167 increased, the luciferase activity simultaneously increased, indicating that Sirt6 suppression promoted Smad3 downstream signaling, consistent with the results shown in Figures 3(d) and 3(e). Considering that Sirt6 deacetylates the conserved lysine residues K333 and K378 in Smad3, as reported by Zhong et al. [23], we constructed lentiviruses with WT-Smad3 or Mut-Smad3 and transduced them into HK2-Smad3^−/−^ cells to generate cell lines stably overexpressing WT-Smad3 or Mut-Smad3. Next, we performed SBE reporter assays using these cell lines. Interestingly, the modulation of luciferase activity by Sirt6 was observed in the WT-Smad3 group but not in the Mut-Smad3 group, demonstrating that Sirt6 also deacetylates Smad3 at lysine 333 and lysine 378 in HK2 cells (Figure 3(f)).

Additionally, it observed that nuclear Smad3 levels were increased upon Sirt6 downregulation in HK2 cells cultured in the Mann medium, whereas these levels were reduced by Sirt6 overexpression in HG-induced HK2 cells (Figure 3(g)). These results suggest that Smad3 deacetylation prevents it from translocating into the nucleus, necessary for downstream signaling. We also measured nuclear Smad3 levels in the kidney cells of db/db or db/+ mice injected with the si-NC or si-Sirt6 lentivirus. The mice treated with si-Sirt6 showed higher nuclear Smad3 levels in both the db/db and db/+ mice (Figure 3(h)). The Smad3 nuclear localization performed by IF showed similar results (Figure 3(i)).

Together, our findings show that Sirt6 deacetylates Smad3 at lysine residues K333 and K378 and reduces the translocation of Smad3 into the nucleus, resulting in the attenuation of downstream signaling and the prevention of kidney injury induced by type II DN in vitro and in vivo.

### 3.4. FOXO3a Inhibits the EMT in HK2 Cells by Binding to the Sirt6 Promoter and Increasing Its Expression

**Figure 4:**
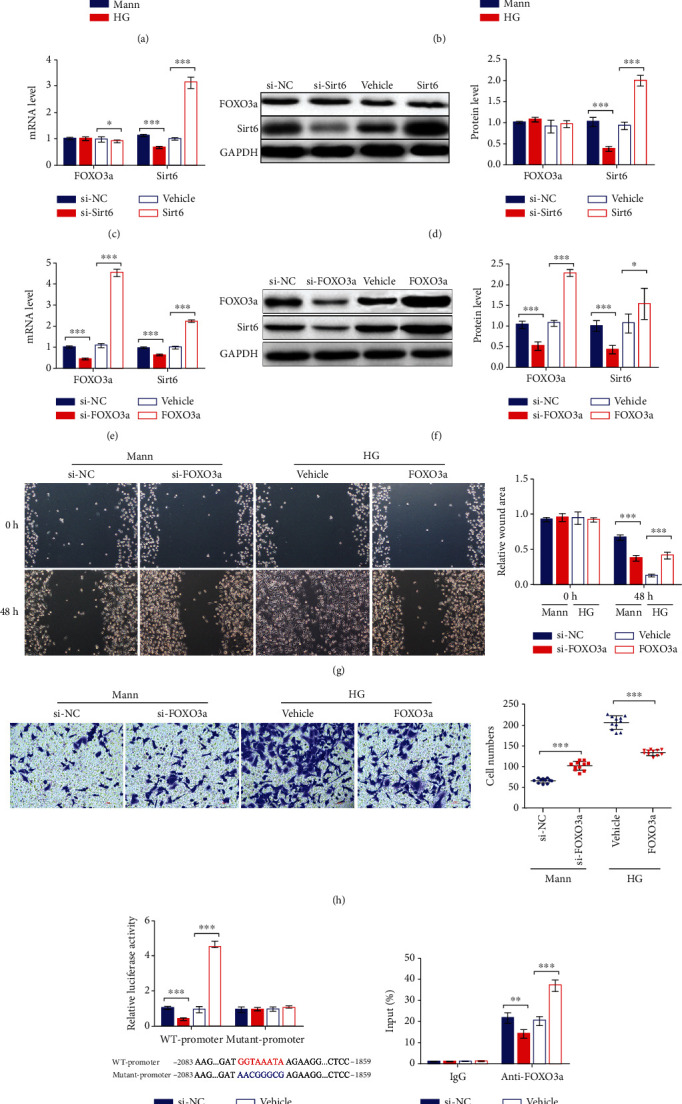
FOXO3a regulates the EMT process in HK2 cells by binding to the Sirt6 promoter and increasing its expression. (a, b) HK2 cells were cultured in the HG medium for 72 h. (a) FOXO3a and Sirt6 mRNA expression analyzed by qPCR. (b) FOXO3a and Sirt6 protein expression assessed by western blot analysis. (c–f) HK2 cells cultured in the Mann medium were transfected with si-Sirt6 (c, d) or si-FOXO3a (e, f) to knock down Sirt6 or FOXO3a, respectively, or were transfected with si-NC for 72 h. HG-induced HK2 cells were transfected with Sirt6 (c, d) or FOXO3a (e, f) overexpression vectors to increase Sirt6 or FOXO3a expression, respectively, or were transfected with the vehicle for 72 h. (c, e) FOXO3a and Sirt6 mRNA expression analyzed by qPCR. (d, f) FOXO3a and Sirt6 protein expression assessed by western blot analysis. (g) Representative images of cell migration and graphs showing the wound area quantification for HK2 cells. After 48 h of transfection, wounds were generated with a 200 *μ*l pipette tip and cultured for another 48 h. HK2 cells transfected with si-NC or si-FOXO3a were cultured in the Mann medium, and cells transfected with the vehicle or the FOXO3a overexpression vector were cultured in the HG medium. (h) Representative images of Transwell assay and graphs showing migrated cells. After 24 h of seeding, HK2 cells were transfected with si-NC or si-FOXO3a cultured in the Mann medium and transfected with the vehicle or the FOXO3a overexpression vector cultured in the HG medium. (i) The luciferase activity of promoter dual-luciferase assays. The -2083 to -1859 region of the Sirt6 promoter was inserted into the pGL3 vector. HK2 cells cultured in the Mann medium were cotransfected with pGL3, pRL-TK, and si-FOXO3a or FOXO3a for 48 h. (j) The ChIP RT-qPCR assay results were used to assess the enrichment of FOXO3a in the Sirt6 promoter region in HK2 cells cultured in the Mann medium for 72 h. The data are presented as the means ± SD. *n* = 3 experiments in (a–j). ^∗^*p* < 0.05, ^∗∗^*p* < 0.01, and ^∗∗∗^*p* < 0.01.

Subsequently, we wanted to assess how Sirt6 is regulated during the EMT in HK2 cells. Inspired by the work of Dong and colleagues, which demonstrated that FOXO3a could be enriched in the Sirt6 promoter region and promote its transcription in the MV3 melanoma cell line [27], we hypothesized that Sirt6 regulation during the EMT in HK2 cells is associated with FOXO3a. To test our hypothesis, we first tested FOXO3a levels in HK2 cells cultured in the HG medium. Interestingly, we observed that FOXO3a mRNA levels in HG-induced HK2 cells were decreased compared to that observed in cells cultured in the Mann medium, with an associated reduction in FOXO3a protein levels (Figures 4(a) and 4(b)). Moreover, we evaluated the changes in FOXO3a expression concerning changes in Sirt6 levels caused by si-Sirt6 treatment or Sirt6 overexpression. The qPCR and western blot results showed little alteration in FOXO3a in response to Sirt6 upregulation or downregulation (Figures 4(c) and 4(d)). Besides, we altered FOXO3a expression through si-FOXO3a or by FOXO3a overexpression in HK2 cells. The results showed that Sirt6 levels decreased when FOXO3a was inhibited by si-FOXO3a, while higher Sirt6 expression was observed in response to FOXO3a overexpression (Figures 4(e) and 4(f)). These results indicated that FOXO3a functions upstream of Sirt6.

Additionally, we performed a wound healing assay and Transwell assay to evaluate the influence of FOXO3a on the EMT in HK2 cells. In Mann-induced HK2 cells, we observed that migration was promoted in the group treated with si-FOXO3a based on the reduced relative wound area (Figure 4(g)). However, the migration of HK2 cells was suppressed when FOXO3a was overexpressed in the HG-induced group, with a slight increase in the relative wound area (Figure 4(g)). Similarly, the Transwell assay results showed that the migration of HK2 cells increased in FOXO3a suppressed HK2 cells and decreased in FOXO3a overexpressing HK2 cells (Figure 4(h)). These migration results indicated that FOXO3a inhibits EMT in HK2 cells.

Subsequently, we performed dual-luciferase assays to confirm the binding between FOXO3a and the -2128 to -1514 bp region of the Sirt6 promoter as previously described by Dong et al. [27]. In the presence of si-FOXO3a, luciferase activity was inhibited (Figure 4(i)), while that observed in cells cotransfected with the FOXO3a overexpression plasmid was greatly increased (Figure 4(i)). On the other hand, the luciferase activity of HK2 cells expressing the mutant Sirt6 promoter displayed no significant change, whether FOXO3a levels are low or high (Figure 4(i)). Next, we performed chromatin immunoprecipitation PCR (ChIP-PCR) assays to confirm further the binding of FOXO3a to the Sirt6 promoter in HK2 cells. The percent input was decreased in cells treated with si-FOXO3a, whereas enhanced binding was observed in cells overexpressing FOXO3a (Figure 4(j)).

Together, the above results showed that FOXO3a inhibited the EMT induced by the HG medium in HK2 cells through binding to the Sirt6 promoter and increasing its expression. Sirt6 downregulation in HG-induced HK2 cells was also associated with reduced FOXO3a expression.

### 3.5. FOXO3a Protects Renal Tubules from Tubular Injury in db/db Mice

**Figure 5:**
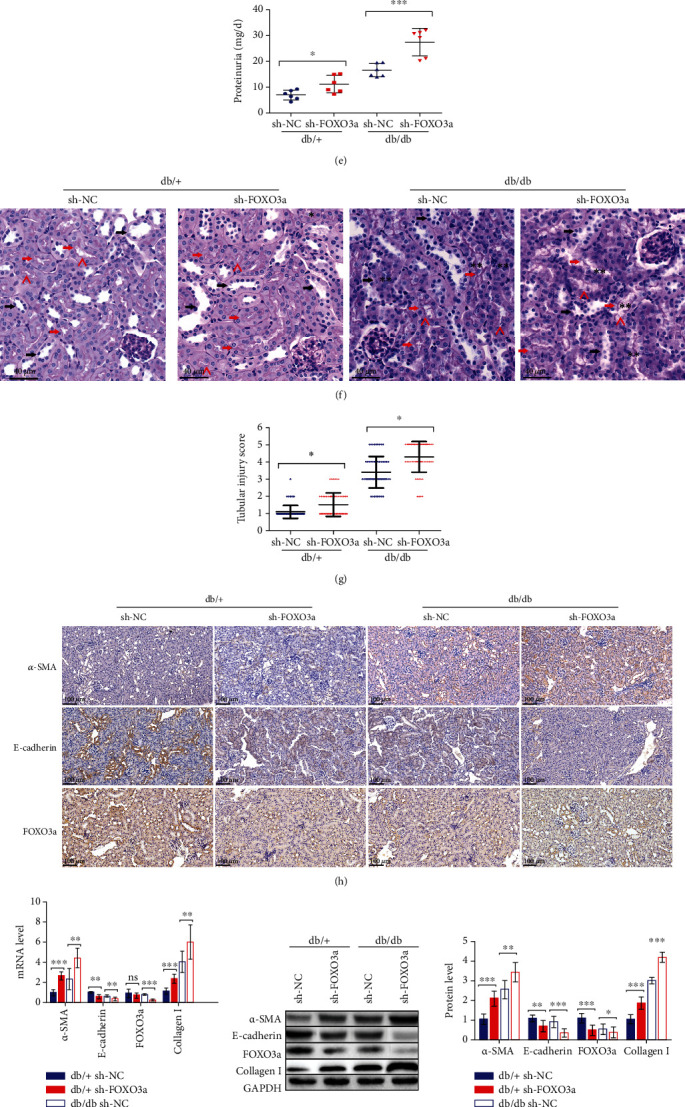
Downregulation of FOXO3a leads to the deterioration of kidney injury in db/db mice. (a, b) Protein and mRNA were extracted from the kidneys of 20-week-old db/db and db/+ mice. (a) FOXO3a mRNA expression analyzed by qPCR. (b) FOXO3a protein expression assessed by western blot analysis. (c–j) The db/db or db/+ mice used in all experiments were 20 weeks old and were infected with sh-NC or sh-FOXO3a lentiviruses in the kidneys at 18 weeks old to knock down FOXO3a expression in vivo. (c–e) Graphs showing ACR, BUN, and proteinuria as analyzed by the ELISA kit. (f) Representative images of PAS staining of kidneys from mice. Scale bar, 40 *μ*m. (g) Scoring of tubular injury. Proximal and distal tubules are marked with red and black arrows, respectively. “^” represents the brush border of proximal tubules. “^∗^” represents the cellular debris in the lumen of proximal tubules. “^∗∗^” represents the disorder proximal tubular structure, respectively. (h) IHC analysis of *α*-SMA, E-cadherin, and FOXO3a expression in kidney tissue. (i) *α*-SMA, E-cadherin, FOXO3a, and Collagen I mRNA expression analyzed by qPCR. (j) Protein expression of *α*-SMA, E-cadherin, FOXO3a, and Collagen I assessed by western blot analysis. (k) KIM-1 and NGAL mRNA expression analyzed by qPCR. (l) Protein expression of KIM-1 and NGAL assessed by western blot analysis. The data are presented as the means ± SD. *n* = 6 experiments in (a–e) and (h–l). *n* = 60 experiments in (g). ^∗^*p* < 0.05, ^∗∗^*p* < 0.01, and ^∗∗∗^*p* < 0.01.

To provide further insights into the signaling mechanism involved in the FOXO3a-mediated EMT process, we evaluated FOXO3a expression in the kidneys of db/db and db/+ mice. The qPCR and immunoblotting results showed that FOXO3a expression in the kidneys of db/db mice was decreased compared with that observed in the kidneys of db/+ mice, indicating that FOXO3a may affect the EMT in vivo (Figures 5(a) and 5(b)) (*p* = 0.0408 in Figure 5(a)).

To test this possibility, we generated FOXO3a knockdown db/db and db/+ mice by injecting them with lentiviruses containing sh-FOXO3a. First, we observed a significant increase in ACR, BUN, and proteinuria levels when db/db or db/+ mice were treated with sh-FOXO3a (Figures 5(c)–5(e)), suggesting that the kidney functions were injured when FOXO3a expression was inhibited, potentially due to the tubular injury caused by FOXO3a reduction.

Then, in the kidneys of db/db mice injected with the sh-FOXO3a lentivirus, we observed the disorder proximal tubular structure and cellular debris in the lumen as well as the loss of the brush border in some proximal tubules, indicating severe tubular damage, as analyzed by PAS staining (Figures 5(f) and 5(g)). Furthermore, IHC assays were performed to evaluate the EMT-associated markers and FOXO3a levels in kidneys. First, we observed that compared to the db/+ mice, *α*-SMA expression was elevated, and E-cadherin and FOXO3a levels were decreased in db/db mice injected with the sh-NC lentivirus (Figure 5(h)). Subsequently, we also observed that in the sh-FOXO3a lentivirus-induced groups, both the db/db and db/+ mice showed increasing *α*-SMA expression and decreasing E-cadherin expression compared to the sh-NC groups (Figure 5(h)). Consistent with the IHC findings, the qPCR and immunoblotting results showed higher *α*-SMA levels and lower E-cadherin levels in the sh-FOXO3a lentivirus groups, indicating that FOXO3a downregulation exacerbated the EMT in renal tubules (Figures 5(i) and 5(j)). We also found that Collagen I expression was increased in both the db/db and db/+ mice treated with sh-FOXO3a (Figures 5(i) and 5(j)). Besides, the markers of tubular damage were analyzed by qPCR and immunoblotting. We found a higher level of KIM-1 and NGAL in mice treated with sh-FOXO3a, no matter in db/db mice or db/+ mice (Figures 5(k) and 5(l)), indicating an exasperated tubular injury in sh-FOXO3a groups consisting with previous results.

Together, our findings demonstrated that FOXO3a could halt the EMT process and tubular injury in the kidneys of db/db mice, mitigating kidney injury.

## 4. Discussion

Mounting evidence has demonstrated the important role of epigenetic modifications in regulating renal function [35, 36], although understanding the functions of deacetylase in the kidney remains limited. Sirt6 is a type of deacetylase that is important in the EMT process in different cells or diseases through different pathways, such as in promoting EMT by inhibiting KLF4 in non-small-cell lung cancer [37], by stimulating the autophagic degradation of E-cadherin in hepatocellular carcinoma [38], or by altering HIF-*α* activity in papillary thyroid cancer [39]. Additionally, some studies have shown that Sirt6 attenuates the EMT process in some diseases, such as suppressing the TGF-*β*1/Smad3 pathway and c-Jun in an asthma model [40] or inactivating the TGF-*β*1/Smad2 pathway in bronchial epithelial cells [41]. Furthermore, Sirt6 has also been reported to display a protective role in renal injury. For example, older Sirt6 knockout mice showed improved renal fibrosis [42], and Sirt6 deficiency in kidneys exacerbated podocyte injury and proteinuria in DN [24]. Considering the role of Sirt6 in EMT and renal fibrosis in DN, we hypothesized that it might ameliorate renal fibrosis by inhibiting EMT and tubular injury. Therefore, we characterized the expression of Sirt6 in HK2 cells induced by HG treatment, which simulated a diabetic environment in vitro [24]. Sirt6 expression was observed in HG-induced HK2 cells with a notable change in EMT marker expression. Subsequent analyses showed that Sirt6 regulates the EMT process in HK2 cells. Furthermore, we generated Sirt6 knockdown db/db or db/+ mice and observed that Sirt6 deficiency exacerbated EMT and tubular injury in the kidneys of both the db/db and db/+ mice, indicating that Sirt6 protects kidneys from injury induced by DN in vivo.

Emerging evidence has emphasized the importance of Smad3, a major downstream mediator of TGF-*β*, in the TGF-*β*-induced EMT process and renal tubular fibrosis [32]. Smad3 is regarded as being involved in pathogenic processes by suppressing fibrosis in kidney disease upon Smad3 deletion [14], such as in DN [43]. Also, Zhong et al. showed that Sirt6 suppresses Smad3 activity by deacetylating it at key lysine residues (K333 and K378) in hepatic stellate cells to attenuate liver fibrosis [23]. Considering the results of these previous studies, in the present study, we investigated the mechanism associated with the role of Sirt6 in inhibiting EMT and tubular injury in DN. Our results confirmed that Sirt6 was also able to deacetylate Smad3 in HK2 or kidney tissue at K333 and K378, which significantly inhibited its activity from promoting downstream gene expression. Some studies have shown that Smad2 acetylation is required for its nuclear accumulation in epithelial cells [44, 45]. Based on the high homology between Smad2 and Smad3, we measured the nuclear accumulation of Smad3, observing that decreased Smad3 acetylation reduced its nuclear localization, which may contribute to its reduced transcriptional activity.

FOXO3a is an important member of the FOXO family involved in modulating the progression of several different diseases, such as cancer and metabolism diseases [27]. Notably, FOXO3a is also a crucial regulator in cancer EMT [46] and significantly influences kidney diseases. The accumulation and activation of FOXO3a aggravate oxidative stress-induced podocyte injury [47]. However, Peng et al. hypothesized that FOXO3a could increase LC3B expression, suppressing renal tubular epithelial cell apoptosis, necrosis, and injury [48]. Lin and colleagues demonstrated that FOXO3a, mediated by Sirt3, suppresses the EMT in renal fibrosis vascular pathology [49]. Based on previous studies showing direct binding between FOXO3a and the Sirt6 promoter in melanoma [27], we assessed whether FOXO3a directly controls Sirt6 levels. FOXO3a levels in HG-induced HK2 cells and db/db mice were dramatically decreased, explaining the observed reduction in Sirt6 levels. Furthermore, FOXO3a knockdown db/db or db/+ mice were generated to study the role of FOXO3a in the diabetes-induced EMT process in vivo. The results showed similar trends to EMT and tubular injury in Sirt6 knockdown mice, while a more severe pathology was observed in sh-FOXO3a groups, indicating the protective role of FOXO3a in diabetes-induced kidney injury.

## 5. Conclusion

Taken together, the results of our present study, for the first time, elucidated the mechanism by which Sirt6 regulates EMT and tubular injury in DN. Sirt6 inhibits Smad3 nuclear accumulation by promoting its deacetylation, which results in the suppression of downstream signaling and attenuates kidney injury in DN. Furthermore, FOXO3a can also protect against kidney injury in DN by binding to the promoter of Sirt6 and enhancing its expression. Thus, pharmacological targeting of FOXO3a-mediated Sirt6/Smad3 signaling pathways may provide a novel strategy for treating type II DN.

## Data Availability

The data used to support the findings of this study are available from the corresponding author upon request.

## Abbreviations

DN: Diabetic nephropathy
EMT: Epithelial-mesenchymal transition
HG: High glucose
Mann: Mannitol
siRNAs: Small interfering RNAs
shRNA: Small hairpin RNA
SBE: Smad-binding element
IHC: Immunohistochemistry
PAS: Periodic acid-Schiff
ChIP: Chromatin immunoprecipitation
qPCR: Quantitative reverse transcription PCR
co-IP: Coimmunoprecipitation
PMSF: Phenylmethylsulfonyl fluoride
ACR: Albumin and creatinine ratio
BUN: Blood urea nitrogen
ChIP-PCR: Chromatin immunoprecipitation PCR
IF: Immunofluorescence
KIM-1: Kidney injury molecule-1
NGAL: Neutrophil gelatinase-associated lipocalin.

## Supplementary Materials

Supplementary Materials Supplementary Figure 1: fasting blood glucose level measurement. (a, b) Fasting blood glucose levels in db/db mice and db/+ mice measured weekly. (a) The change of fasting blood glucose levels during 8~18 weeks. (b, c) The change of fasting blood glucose levels before and after sh-Sirt6 (b) and sh-FOXO3a (c) lentivirus injection. The data are presented as the means ± SD. *n* > 10 experiments in (a). *n* = 6 experiments in (b, c). ^∗^*p* < 0.05, ^∗∗^*p* < 0.01, and ^∗∗∗^*p* < 0.01.
