# Supplementary material for: FOXO3a Protects against Kidney Injury in Type II Diabetic Nephropathy by Promoting Sirt6 Expression and Inhibiting Smad3 Acetylation
Source: Oxid Med Cell Longev. 2021 May 26;2021:5565761. doi: 10.1155/2021/5565761 (PMC8172321; doi:10.1155/2021/5565761)
Supplement: Supplementary Materials — Supplementary Figure 1: fasting blood glucose level measurement. (a, b) Fasting blood glucose levels in db/db mice and db/+ mice measured weekly. (a) The change of fasting blood glucose levels during 8~18 weeks. (b, c) The change of fasting blood glucose levels before and after sh-Sirt6 (b) and sh-FOXO3a (c) lentivirus injection. The data are presented as the means ± SD. n > 10 experiments in (a). n = 6 experiments in (b, c). ∗p < 0.05, ∗∗p < 0.01, and ∗∗∗p < 0.01. [file 5565761.f1.zip › Table S1.docx]

Table S1: The sequences of si-RNA and primers

| Name | Sequences (5’-3’) |
| --- | --- |
| si-Sirt6 | ACGGGAACAUGUUUGUGGAAG |
| si-FOXO3a | AGCCCUGCCAAGCCCUCGGGG |
| si-NC | UUCUCCGAACGUGUCACGU |
| sh-Sirt6 | AAGAATGTGCCAAGTGTAAGA |
| sh-FOXO3a | AATGTGACATGGAGTCCATTAT |
| sh-NC | ATCCGTCCGAACGTAAGTCAA |
| Sirt6-F | CCGGAATTCATGTCGGTGAATTACGCGGCGGC |
| Sirt6-R | CGCGGATCCTTAACTGGGGACCGCCTTGG |
| FOXO3a-F | GAATTCATGGCAGAGGCACCGGCTTCCCCG |
| FOXO3a-R | GGATCCTCAGCCTGGCACCCAGCTCTGAGAT |
| WT-Smad3-F | GAATTCATGGAGCTGTGTGAGTTCGCCTT |
| WT-Smad3-R | GGATCCCTAAGACACACTGGAACAGCG |
| Sirt6-Promoter-F | CCGGCTAGCGGGTAATAAGACACCCAACAGAGG |
| Sirt6-Promoter-R | CCGCTCGAGGTAATGGTGACATGGTGTGGTTG |
| qPCR-Sirt6-F | CTCGAAGTGGAGCTGGACC |
| qPCR-Sirt6-R | TCCTCGGGGATCATGGAGTC |
| qPCR-FOXO3a-F | ACGTCTTCAGGTCCTCCTGTT |
| qPCR-FOXO3a-R | GGGGAAGCACCAAAGAAGAGAG |
| qPCR-E cadherin-F | GCTGGACCGAGAGAGTTTCC |
| qPCR-E cadherin-R | CAAAATCCAAGCCCGTGGTG |
| qPCR-TGFβ-F | ACAGCAACAATTCCTGGCGA |
| qPCR-TGFβ-R | CCGTTGATGTCCACTTGCAG |
| qPCR-αSAM-F | GGTGCTGTCTCTCTATGCCT |
| qPCR-αSAM-R | AAGGAATAGCCACGCTCAGT |
| qPCR-GAPDH-F | CTCTGCTCCTCCTGTTCGAC |
| qPCR-GAPDH-R | GCGCCCAATACGACCAAATC |
