# Supplementary figures and images for: FOXO3a Protects against Kidney Injury in Type II Diabetic Nephropathy by Promoting Sirt6 Expression and Inhibiting Smad3 Acetylation
Source: Oxid Med Cell Longev. 2021 May 26;2021:5565761. doi: 10.1155/2021/5565761 (PMC8172321; doi:10.1155/2021/5565761)

**a**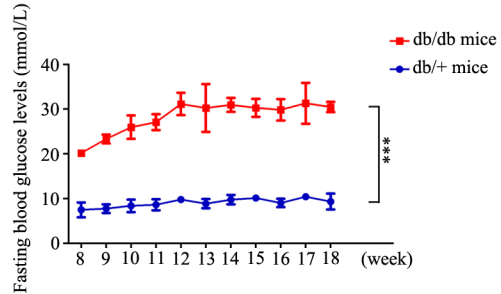**b**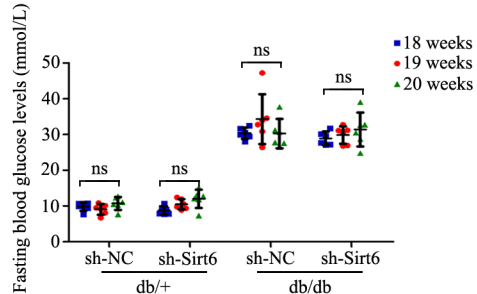**c**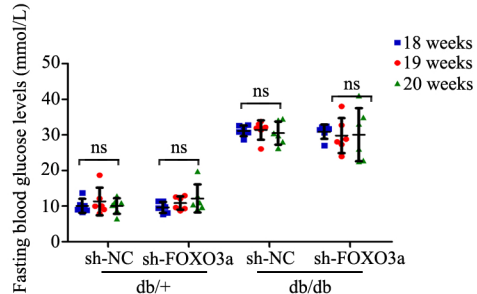

Supplement: Supplementary Materials — Supplementary Figure 1: fasting blood glucose level measurement. (a, b) Fasting blood glucose levels in db/db mice and db/+ mice measured weekly. (a) The change of fasting blood glucose levels during 8~18 weeks. (b, c) The change of fasting blood glucose levels before and after sh-Sirt6 (b) and sh-FOXO3a (c) lentivirus injection. The data are presented as the means ± SD. n > 10 experiments in (a). n = 6 experiments in (b, c). ∗p < 0.05, ∗∗p < 0.01, and ∗∗∗p < 0.01. [file 5565761.f1.zip › Figure S1.pdf]
